# The value and limitations of the Oxford Community Stroke Project classification in the hyperacute phase of cerebral infarction

**DOI:** 10.3389/fneur.2025.1731686

**Published:** 2026-01-16

**Authors:** Yuanjia Miao, Xiaofeng Li, Shaoshuai Wang, Changxin Li

**Affiliations:** 1 Clinical College, Shanxi Medical University, Taiyuan, Shanxi, China; 2 Department of Neurology, First Hospital of Shanxi Medical University, Taiyuan, Shanxi, China

**Keywords:** acute ischemic stroke, neuroimaging, OCSP classification, reperfusion therapy, symptomatic intracranial hemorrhage

## Abstract

Originating from a large-scale community-based screening program in the United Kingdom, the Oxford Community Stroke Project (OCSP) classification has become a pivotal tool for categorizing ischemic stroke and guiding clinical decision-making since its inception. In the context of reperfusion therapy for hyperacute cerebral infarction, the integration of the OCSP classification with clinical functional assessment and neuroimaging enables clinicians to rapidly identify stroke subtypes, tailor recanalization strategies, and prognosticate associated complications. This review summarizes the application of the OCSP classification in the diagnosis and management of hyperacute ischemic stroke and discusses how to optimize its utility to provide precision treatment in the modern era of neuroimaging.

Ischemic stroke is defined as localized ischemic injury to brain tissue resulting from a sudden interruption of cerebral blood flow. The hyperacute phase represents the critical therapeutic window; during this period, the timely removal of arterial obstruction, achievement of recanalization, and restoration of cerebral perfusion are key strategies for improving patient prognosis (1). However, decision-making in the hyperacute phase is inherently complex. Clinicians must accurately assess the risk-benefit ratio and determine the optimal treatment regimen within a strictly limited timeframe. Furthermore, traditional assessment tools often prioritize pathogenesis, making it difficult to provide rapid, precise guidance during the hyperacute phase (2).

The Oxford Community Stroke Project (OCSP) classification system originated from community-based population studies in the 1980s. This system categorizes ischemic stroke into four major subtypes based on neurological examination: Total Anterior Circulation Infarct (TACI), Partial Anterior Circulation Infarct (PACI), Posterior Circulation Infarct (POCI), and Lacunar Infarct (LACI) (3). The OCSP classification determines stroke type through straightforward neurological signs, thereby avoiding immediate reliance on complex imaging while maintaining a strong correlation with underlying cerebrovascular pathology. With its extensive clinical adoption, the OCSP classification has evolved into an effective instrument for guiding therapeutic strategies and assessing the risk of stroke-associated complications. This paper reviews the characteristics of OCSP subtypes and

their application in hyperacute cerebral infarction, while exploring avenues to further enhance their clinical value in the current neuroimaging era.

A comprehensive literature search was performed across three databases (PubMed, Embase, and the Cochrane Library) for studies published from 1990 through October 1, 2025. The search strategy combined the use of medical subject headings (MeSH) and keywords. The search terms included “Oxford Community Stroke Project classification” and its subtypes (“total anterior circulation infarct,” “partial anterior circulation infarct,” “posterior circulation infarct,” and “lacunar infarct”), “ischemic stroke,” and “reperfusion therapy” (incorporating “intravenous thrombolysis” and “endovascular treatment”). Literature screening focused on the clinical utility and limitations of the OCSP classification in the hyperacute phase of ischemic stroke, prioritizing randomized controlled trials (RCTs), meta-analyses, and high-quality cohort studies.

## 1 Introduction to the OCSP classification

The OCSP classification system categorizes ischemic stroke into four subtypes based on clinical presentation: total anterior circulation infarct (TACI), partial anterior circulation infarct (PACI), lacunar infarct (LACI), and posterior circulation infarct (POCI). TACI is typically associated with occlusion of the middle cerebral artery (MCA) main stem or the internal carotid artery (ICA). It manifests as a clinical triad: higher cortical dysfunction, homonymous hemianopia, and contralateral motor or sensory deficits. PACI predominantly results from occlusion of MCA branches and presents with only one or two components of the TACI triad. LACI primarily affects the territories of perforating arteries. It is characterized by distinct clinical syndromes—pure motor stroke, pure sensory stroke, sensorimotor stroke, or ataxic hemiparesis—with lesion diameters typically measuring less than 1.5 cm. POCI involves the vertebrobasilar arterial system, exhibiting diverse clinical features such as vertigo, ataxia, and cranial nerve palsies (4). Given the strong correlation between OCSP subtypes and underlying cerebrovascular pathology, this system facilitates the inference of etiology during the hyperacute phase. Current evidence highlights distinct etiological profiles for proximal vs. distal occlusions. The TACI subtype, often caused by ICA or proximal MCA occlusion, is frequently attributed to large-artery atherosclerosis. Conversely, the PACI subtype, arising from distal MCA occlusion, is more commonly associated with embolic mechanisms (5). The POCI subtype, resulting from vertebrobasilar involvement, is frequently linked to arterial dissection or perforator disease, whereas embolism is a relatively less common mechanism (6). Finally, for the LACI subtype, which involves perforating arteries, the pathogenesis necessitates differentiation between intrinsic small vessel disease (e.g., lipohyalinosis of arterioles, endothelial dysfunction) and non-small vessel pathologies, such as large artery atherosclerosis or cardioembolism (7). Beyond delineating vascular pathology, these subtypes are also intrinsically linked to distinct prognostic profiles, including the risk of hemorrhagic transformation and systemic complications. To provide a comprehensive overview, we summarize the typical vascular pathology, pathogenesis, and complication risks of each OCSP subtype in Table 1.

**Table 1:** Clinical characteristics, pathogenesis, and complication risks by OCSP subtype.

**Feature**	**TACI**	**PACI**	**POCI**	**LACI**
Typical vascular pathology	Proximal large vessel occlusion (typically involving the terminal internal carotid artery or the MCA M1 segment)	Distal or branch occlusion (commonly involving the distal MCA or its branches)	Vertebrobasilar artery territory	Perforating artery or occult large vessel occlusion
Pathogenesis	Predominantly large-artery atherosclerosis; embolism is also a potential cause	Predominantly associated with embolic mechanisms	Common etiologies include arterial dissection or perforating artery pathology	Traditionally attributed to small-vessel lipohyalinosis; however, large-vessel stenosis or occlusion is present in some cases
Risk of sICH after reperfusion therapy	High	Moderate	Low	Low
Systemic complications	High frequency of systemic complications: 1. Urinary incontinence: high risk due to damage to cortical centers 2. Pneumonia: high risk secondary to immobility (bedridden state) and dysphagia	Relatively fewer systemic complications	High risk of pneumonia secondary to immobility and dysphagia	Low frequency

## 2 Application of the OCSP classification in the diagnosis of hyperacute ischemic stroke

Currently, the diagnosis of ischemic stroke relies primarily on neuroimaging. However, during the hyperacute phase, constraints related to imaging resolution and time windows mean that computed tomography (CT) or magnetic resonance imaging (MRI) may fail to clearly visualize the culprit lesion. Given the established concordance between the OCSP classification and neuroimaging findings, this clinical system facilitates rapid diagnosis and stratification, thereby offering preliminary guidance for reperfusion therapy selection.

Following the initial proposal of the OCSP classification, early validation studies examined its concordance with radiographic findings, revealing a significant correlation between OCSP subtypes and both infarct location and volume. In a prospective study involving 1,012 patients with ischemic stroke, Mead et al. (8) analyzed this clinicoradiological correlation. Their findings demonstrated that in three-quarters of the cohort, the infarct location and volume were accurately predicted based solely on the clinical classification. Similarly, a Brazilian study reported high overall concordance (88.1%) between OCSP classification and neuroimaging, although the agreement for the LACI subtype was notably lower, at approximately 65.5% (9). The reduced concordance observed in the LACI subtype may be attributed to limited imaging resolution and the lack of specificity in lacunar syndromes, which can frequently be clinically indistinguishable from PACI presentations. Recent evidence suggests that integrating the National Institutes of Health Stroke Scale (NIHSS) score as an adjunctive tool can enhance the diagnostic accuracy of the LACI subtype (10).

## 3 Application of the OCSP classification in reperfusion therapy for ischemic stroke

Intravenous thrombolysis (IVT) and endovascular treatment (EVT) currently represent the cornerstones of hyperacute reperfusion therapy for ischemic stroke (11). This section reviews the specific utility and predictive value of the OCSP classification within the scope of these two therapeutic modalities.

### 3.1 Application of the OCSP classification in intravenous thrombolysis

Since the seminal NINDS trial first demonstrated in 1995 that intravenous administration of tissue plasminogen activator (t-PA) within 3 h of onset significantly improves clinical outcomes in ischemic stroke (12), substantial progress has been made regarding the extension of the therapeutic window and the application of IVT in minor stroke. However, given the significant heterogeneity in reperfusion responses across different OCSP subtypes, traditional homogenous screening strategies based solely on a uniform time window no longer suffice to meet the demands of precision medicine. Recent research has focused on enhancing thrombolytic efficacy through precise patient selection. This section critically synthesizes the latest evidence regarding IVT decision-making across different subtypes, aiming to provide clinicians with a stratified therapeutic perspective based on classification.

#### 3.1.1 Anterior circulation infarction (TACI/PACI): extending the therapeutic window via clinical-imaging mismatch

For anterior circulation subtypes, the primary challenge in clinical IVT lies in accurately identifying the ischemic penumbra when the time of onset is unclear (e.g., wake-up strokes) or exceeds standard windows. While the OCSP classification rapidly indicates the anatomical territory of vascular involvement, it lacks the capacity to precisely quantify clinical severity or definitive infarct volume. Consequently, integrating OCSP classification with the NIHSS score and neuroimaging becomes pivotal for optimizing IVT strategies.

To overcome the limitations of isolated clinical assessment, Dávalos et al. (13) pioneered the concept of “clinical-diffusion mismatch” (CDM), thereby establishing a critical link between clinical symptomatology and histopathological status. Prosser et al. (14) further validated the correlation between CDM and perfusion-diffusion (PWI-DWI) mismatch. This retrospective study demonstrated that, following the exclusion of posterior circulation strokes, the presence of a severe anterior circulation deficit (NIHSS ≥8) alongside a restricted ischemic core on diffusion-weighted imaging (DWI volume <25 ml) predicts the existence of the ischemic penumbra with high specificity. Effectively, this approach utilizes the NIHSS to complement the OCSP classification, leveraging the discrepancy between clinical severity and tissue necrosis volume to precisely target salvageable brain tissue.

Considering the accessibility and time constraints associated with advanced imaging, Wu et al. (15) retrospectively validated the feasibility of a CT-based screening strategy. Their study revealed that for anterior circulation stroke patients with an NIHSS ≥8, a high Alberta Stroke Program Early CT Score (ASPECTS ≥9)—indicating minimal early ischemic changes on CT—suggests that the core infarct has not yet extensively formed despite severe clinical symptoms. Patients exhibiting this “clinical-CT mismatch” demonstrated superior functional recovery following IVT. Recent meta-analyses have further corroborated the safety of this assessment strategy: in populations with wake-up strokes or unknown onset times, IVT administration following screening via CT (to exclude large infarcts) combined with clinical assessment did not increase the risk of symptomatic intracranial hemorrhage (sICH) and significantly improved functional outcomes (16).

In summary, for TACI and PACI subtypes, the OCSP classification should not be regarded as a solitary decision-making endpoint but rather as an anatomical anchor for clinical assessment. By integrating it with NIHSS scoring and neuroimaging, clinicians can more precisely delineate the “tissue window,” thereby expanding the population benefiting from reperfusion therapy without compromising safety. However, it is crucial to acknowledge that while current evidence is promising, it predominantly stems from retrospective analyses; consequently, further validation through prospective trials remains necessary.

#### 3.1.2 Posterior circulation infarction (POCI): overcoming imaging blind spots and the limitations of assessment tools

Decision-making regarding IVT for posterior circulation infarction presents significantly greater challenges than for anterior circulation strokes. This complexity stems primarily from the higher ischemic tolerance of the posterior circulation territory and its intricate clinical presentation. Notably, studies indicate that even diffusion-weighted imaging (DWI)—the most sensitive MRI sequence for detecting acute cerebral infarction—retains a substantial false-negative rate in the context of POCI (17, 18). This limited radiological sensitivity, compounded by non-specific clinical manifestations such as vertigo and ataxia, frequently prolongs clinical assessment and delays diagnosis.

Data from a meta-analysis highlight the clinical repercussions of this dilemma: only 5%−19% of patients with POCI receive IVT, a shortfall largely attributable to diagnostic delays that push patients beyond the standard therapeutic time window (19). However, evolving therapeutic paradigms have challenged strict time constraints. A recent RCT demonstrated that IVT confers significant clinical benefit in patients with mild POCI presenting 4.5–24 h after onset, provided they have no contraindications and a pc-ASPECTS of ≥7 (20). This finding establishes an evidentiary basis for extending thrombolysis in the posterior circulation using a tissue-window approach.

Nevertheless, limitations remain regarding patient selection, which relied on the NIHSS. Originally designed with a bias toward anterior circulation symptoms, the NIHSS assigns insufficient weight to posterior circulation signs, such as disequilibrium and diplopia. To address this deficiency and enhance early recognition, researchers have proposed novel assessment scales incorporating specific posterior circulation indicators, aiming to more precisely quantify the clinical characteristics of POCI (21, 22). Moving forward, the validation and implementation of these specialized scales will be pivotal for identifying candidates suitable for thrombolysis and enhancing reperfusion rates in the posterior circulation.

#### 3.1.3 Lacunar infarction (LACI): reconciling pathological heterogeneity with therapeutic efficacy

Clinical decision-making regarding the lacunar infarction (LACI) subtype has long been confounded by the significant heterogeneity of its pathogenesis. This subtype may stem from intrinsic small vessel pathologies, such as lipohyalinosis, or be driven by non-small vessel mechanisms, including microembolism (7). Historically, this pathophysiological uncertainty prompted skepticism regarding the rationale for IVT; concerns centered on the premise that isolated structural lesions of the small vessel wall might not benefit from thrombolysis and could, conversely, increase the risk of hemorrhage.

However, accumulating evidence has since redressed this theoretical misconception. Multiple large-scale clinical trials and registry analyses have substantiated that, despite mechanistic complexity, IVT remains a highly effective therapeutic intervention for LACI patients, offering significant improvements in functional outcomes. Current academic consensus attributes this efficacy primarily to two mechanisms: (1) a substantial proportion of patients presenting with LACI syndrome may actually harbor etiologies involving micro-emboli of cardioembolic or arterio-arterial origin, for which thrombolytic therapy effectively promotes vascular recanalization. (2) Beyond direct thrombolysis, t-PA may exert pleiotropic neuroprotective effects, including improving microcirculatory perfusion within the ischemic penumbra, suppressing local inflammatory responses, and mitigating neuronal injury (23, 24).

Consequently, future research should no longer be confined to validating the efficacy of IVT in LACI. Instead, the focus must shift toward a deeper dissection of LACI pathogenesis, thereby providing more precise guidance for clinical IVT administration.

### 3.2 Application of the OCSP classification in endovascular treatment

Since the turn of the 21st century, EVT—particularly mechanical thrombectomy (MT)—has emerged as a pivotal therapeutic intervention for patients with acute ischemic stroke secondary to large vessel occlusion (LVO) (25–27). In this context, the precise identification and prediction of the ischemic penumbra are critical for optimizing EVT strategies. As a rapid classification tool available in the hyperacute phase, the OCSP system facilitates the prediction of LVO presence and the estimation of penumbral extent, thereby informing decision-making within endovascular treatment algorithms.

#### 3.2.1 Anterior circulation infarction subtypes (TACI/PACI): circumventing complex imaging dependencies

For anterior circulation stroke, the paramount clinical priority is the rapid identification of patients with LVO who are candidates for EVT. Evidence suggests that patients presenting with TACI and PACI phenotypes are highly correlated with MCA occlusion—a condition where mechanical thrombectomy yields superior prognostic outcomes. A retrospective study by Xu et al. demonstrated that within 4.5 h of symptom onset, a predictive accuracy of 94.1% for anterior circulation LVO can be achieved by combining the TACI classification with an ASPECTS ≥6 and an NIHSS ≥8 (28).

This implies that in primary stroke centers lacking multimodal CT capabilities, OCSP classification can serve as a reliable trigger for initiating patient transfer protocols. In the screening of anterior circulation stroke patients within the late time window (6–24 h), trials such as DEFUSE-3 have established the pivotal role of perfusion imaging in assessing the ischemic penumbra. However, the “clinically approximated hypoperfused tissue” (CAT) model proposed by Desai et al. offers a streamlined assessment pathway that obviates the need for advanced perfusion imaging. This study demonstrated that integrating the NIHSS with the ASPECTS allows for a straightforward estimation of hypoperfused tissue volume defined by Tmax >6 s. When compared to the DEFUSE-3 cohort, this approach achieved a sensitivity of 100% and a specificity of 92%, indicating that eligible candidates for EVT are virtually never overlooked (29).

*Post-hoc* analyses of two RCTs, DEVT, and RESCUE-BT, provide further corroboration: for patients with anterior circulation LVO, irrespective of the time window, clinical outcomes guided by basic imaging were not statistically distinguishable from those guided by advanced perfusion imaging (30). Collectively, these findings underscore the clinical efficacy of combining OCSP-based clinical assessment with basic neuroimaging for the efficient stratification of patients likely to benefit from EVT, although validation through further prospective RCTs remains necessary.

#### 3.2.2 Posterior circulation infarction (POCI): precision screening via the tissue window

Decision-making regarding EVT for posterior circulation large vessel occlusion has long been plagued by uncertainty regarding its efficacy. Early landmark randomized controlled trials—specifically the BEST and BASICS studies—failed to demonstrate a significant benefit of EVT over medical management. This was largely because their inclusion criteria did not strictly limit infarct core volume, leading to the recruitment of patients with established, irreversible neuronal injury (31, 32). These setbacks underscored a critical clinical reality: reliance on rigid time windows, without a precise assessment of tissue pathophysiological status, is a primary driver of treatment futility.

A turning point in clinical screening strategies emerged with the introduction of semi-quantitative imaging metrics, such as the pc-ASPECTS and the Pons-midbrain index (33, 34). Leveraging these tools to optimize patient selection, the BAOCHE and ATTENTION trials successfully established a clear benefit for EVT in patients with vertebrobasilar artery occlusion within 24 h of onset (35, 36). This paradigm shift provides compelling evidence that, for the POCI subtype, a precision screening strategy based on the “tissue window” is significantly superior to one based solely on the “time window.”

Currently, the boundaries of therapeutic exploration are extending into the ultra-late window beyond 24 h. Multicenter observational data indicate that patients presenting with severe symptoms but small infarct cores may still derive prognostic benefit from EVT beyond the 24-h mark (37). Conversely, for patients with mild symptoms, indiscriminate intervention has not yielded significant clinical gain (38). In conclusion, therapeutic decision-making for POCI must transcend mechanical temporal limits, shifting instead toward an assessment centered on the interplay between clinical severity and infarct core volume to precisely identify beneficiaries within this complex pathology.

#### 3.2.3 Lacunar infarction (LACI): therapeutic decision-making for low NIHSS scores with large vessel occlusion

In the era of EVT, the LACI subtype presents a unique clinical dilemma: how should clinicians manage patients presenting with mild symptoms despite the presence of a LVO? While LACI is traditionally attributed to small vessel pathology, emerging evidence reveals that a substantial proportion of these patients harbor an underlying LVO (39). This finding mandates a re-evaluation of therapeutic strategies for these so-called “minor strokes.”

Currently, large-scale RCTs defining the optimal reperfusion strategy for this specific cohort are lacking; support for aggressive intervention is primarily driven by concerns regarding the natural history of the condition. Kim et al. (40) identified LVO as an independent risk factor for early neurological deterioration, suggesting that the potential risk of hemodynamic decompensation in LACI patients with concomitant LVO should not be underestimated.

While some studies indicate that carefully selected patients with low NIHSS scores and LVO derive significant functional benefit from EVT (41, 42), others comparing EVT with intravenous thrombolysis report no significant difference in efficacy, noting instead a heightened risk of symptomatic intracranial hemorrhage in the EVT group (43). This discrepancy may be attributed to futile reperfusion in some recanalized patients, potentially stemming from poor pre-procedural collateral circulation and lower ASPECTS scores (44). Consequently, we propose that treatment decisions for patients clinically presenting as LACI with underlying LVO should not rely solely on the presence of an occlusion. Instead, decisions must be guided by refined risk stratification incorporating collateral circulation status and baseline characteristics to ensure a cautious and evidence-based approach to EVT selection.

**Figure 1:**
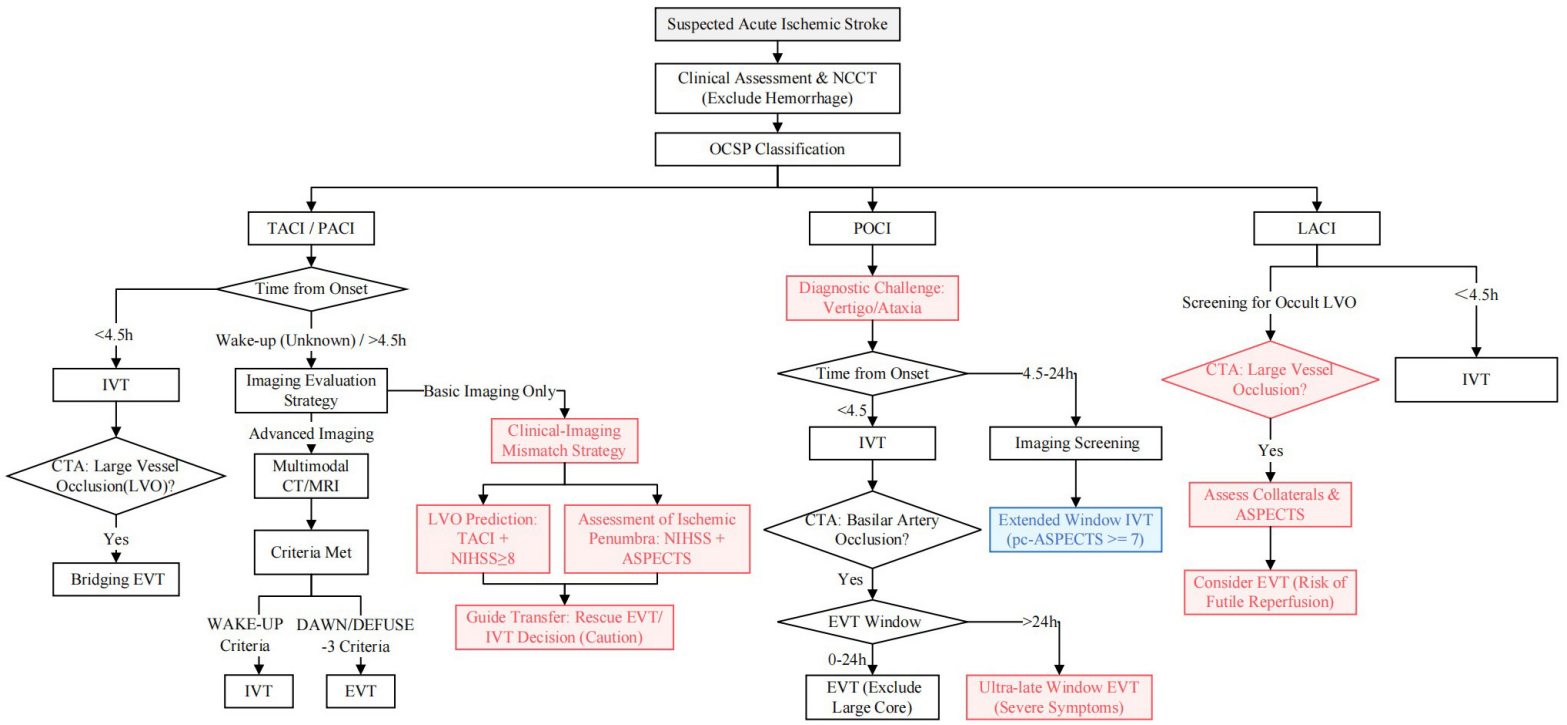
A proposed integrated decision-making framework for hyperacute stroke reperfusion guided by OCSP classification.

Based on the evidence reviewed above, navigating the therapeutic choices for each subtype requires a structured approach. We propose an integrated decision-making framework, illustrated in Figure 1, which synthesizes standard pathways with emerging strategies for diagnostic challenges and extended time windows.

## 4 Application of OCSP classification in complication prediction and risk stratification for ischemic stroke

In the management of hyperacute cerebral infarction, achieving vascular recanalization alone does not mark the conclusion of therapy; rather, the prediction and prevention of complications are paramount for improving long-term patient prognosis. The OCSP classification system serves as a pivotal instrument in this regard. By reflecting not only anatomical characteristics but also correlating highly with infarct volume and affected neural circuits, OCSP classification has become an essential tool for predicting symptomatic intracranial hemorrhage (sICH) and systemic complications.

### 4.1 Symptomatic intracranial hemorrhage (sICH): risk stratification based on anatomical subtypes

sICH is a severe complication following reperfusion therapy, and accurate risk stratification is essential for developing personalized treatment plans. Traditional predictive tools, such as the SITS-sICH score, rely largely on the NIHSS score. However, this scoring system has limitations: it is biased toward language function in the dominant hemisphere. Consequently, patients with right-hemisphere strokes often present with low clinical scores despite having large infarct volumes (45). This “mismatch” between clinical score and pathological volume leads to a discrepancy where, among patients with similar NIHSS scores, those with right-hemisphere non-lacunar strokes have a significantly higher risk of post-thrombolytic hemorrhage than those with left-hemisphere strokes (46). This observation indicates that severity scores alone are insufficient to distinguish between anatomical patterns of involvement. Addressing this issue, Sung et al. (47) showed that incorporating the OCSP classification into risk models, with specific weights for different subtypes, significantly corrects this bias and improves the prediction of sICH. Among the subtypes, TACI consistently carries the highest risk of sICH. In contrast, the POCI subtype shows a relatively lower rate of hemorrhagic transformation, even after thrombolysis. Mechanistically, the relative safety of the POCI subtype may relate to its vascular architecture. First, lesion volumes in posterior circulation infarcts are generally smaller. Second, the brainstem is supplied by small terminal arteries; if a proximal artery is occluded, collateral circulation via the posterior communicating arteries or cerebellar arteries can delay the progression of irreversible ischemia, thereby maintaining vessel wall integrity.

Additionally, biomarkers may provide molecular evidence. Elevated levels of S-100B protein, a marker of blood-brain barrier disruption, are associated with sICH occurrence (48). Potential differences in S-100B expression across OCSP subtypes might explain the heterogeneity in bleeding risk, although further research is needed. In summary, the OCSP classification provides anatomical risk information independent of clinical severity. This stratification suggests that for TACI patients, physicians should implement rigorous monitoring during reperfusion and strictly control modifiable factors to reduce the risk of hemorrhage.

### 4.2 Systemic complications: early warning guided by functional-anatomical correlations

Beyond its utility in assessing hemorrhagic risks, the OCSP classification offers significant predictive value for systemic complications, such as post-stroke pneumonia and urinary incontinence, by delineating specific patterns of neurological deficit. Post-stroke pneumonia represents a leading cause of stroke-related mortality. A large-scale retrospective analysis by Huang et al. (49) revealed that the incidence of pneumonia is significantly lower in patients with LACI compared to those with non-LACI subtypes, particularly TACI and POCI.

The anatomical basis for this disparity lies in the specific structures compromised: non-LACI strokes frequently involve cortical regions or brainstem swallowing centers, resulting in bulbar palsy or severe hemiplegia. These deficits consequently increase the risk of aspiration and attenuate the cough reflex. Conversely, patients with LACI typically preserve functional cough reflexes and maintain greater physical mobility. Similarly, urinary incontinence constitutes a prevalent post-stroke complication. Meta-analyses have identified the TACI subtype as an independent risk factor for post-stroke urinary incontinence. This association is likely attributable to the extensive frontoparietal cortical damage associated with TACI, which disrupts the higher-order neural circuitry governing bladder control (50).

In summary, the OCSP classification serves as a critical early warning indicator for nursing and clinical decision-making. For patients presenting with TACI or POCI, clinical protocols should prioritize the early initiation of dysphagia screening, airway management, and urinary care strategies to mitigate the incidence of systemic complications.

## 5 Integration of OCSP classification with modern neuroimaging

As a stratification system rooted in clinical presentation, the OCSP classification serves as a vital complement to neuroimaging assessments.

Currently, Multi-Detector Computed Tomography (MDCT) protocols—encompassing non-contrast CT (NCCT), dynamic CT perfusion (CTP), and CT angiography (CTA)—remain the standard of care in the emergency department for evaluating vascular status and cerebral perfusion. However, emerging technologies such as Flat Detector Computed Tomography (FD-CT) are reshaping this landscape. Recent pilot studies indicate that an extended multimodal FD-CT approach within the angiography suite can provide a “one-stop-shop” solution, enabling the assessment of intracranial and cervical vasculature immediately prior to thrombectomy. This integration allows for a seamless transition from clinical classification to intervention, significantly reducing door-to-puncture times. In this context, the OCSP classification acts as the initial “triage flag,” guiding patients directly to these advanced imaging pathways (51).

Beyond workflow optimization, the correlation between OCSP subtypes and specific neuroimaging biomarkers offers predictive value. Research indicates that in anterior circulation infarct subtypes, the presence of asymmetrically prominent cortical veins on Susceptibility Weighted Imaging correlates significantly with the extent of the ischemic penumbra, serving as a marker for potential stroke progression (52).

Furthermore, thrombus characteristics, specifically volume and density, critically influence the selection of reperfusion strategies. Studies utilizing semi-automated software analysis of CT and CTA data have demonstrated that a substantial thrombus burden (volume ≥200 mm^3^) is a robust predictor of resistance to recanalization via intravenous thrombolysis alone (53). The integration of these advanced multi-modal imaging parameters with the clinical OCSP classification is, therefore, pivotal for optimizing the diagnosis and management of hyperacute cerebral infarction.

## 6 Limitations and application boundaries of OCSP classification in the modern era

While the OCSP classification provides a foundational framework for rapid stroke categorization, its exclusive reliance on clinical phenotypes presents significant limitations in the era of precision medicine (54). These limitations are increasingly apparent, primarily manifesting as diagnostic uncertainty and insufficient precision in disease assessment.

The diagnostic efficacy of the OCSP classification is intrinsically dependent on the manifestation of typical clinical features. In patients presenting with non-specific symptoms—such as those with posterior circulation strokes manifesting solely as isolated vertigo—reliance on clinical examination alone frequently results in missed diagnoses or misidentification as peripheral vertigo, thereby causing eligible patients to miss the window for thrombolytic therapy. Furthermore, the OCSP classification demonstrates suboptimal specificity in distinguishing stroke mimics. In the absence of neuroimaging confirmation, this limitation poses a risk of unnecessary reperfusion therapy for patients with mimic conditions, thereby increasing iatrogenic risk (55, 56). Additionally, during the hyperacute phase, clinical symptoms often fluctuate due to factors such as collateral compensation and dynamic thrombus migration. This instability leads to temporal inconsistencies in classification. Moreover, the OCSP classification is susceptible to significant subjective bias. A comparative study revealed that the overall accuracy of manual classification by resident physicians was only 72.7%, with significant inter-observer variability observed particularly in differentiating between complex TACI and PACI (57). Such classification drift may obscure the identification of the culprit vessel, subsequently compromising the consistency of reperfusion strategies.

In contrast to modern multimodal neuroimaging, the OCSP classification is incapable of quantifying the volume of the infarct core or the extent of the ischemic penumbra (58–60). For instance, two patients classified with the same POCI subtype may exhibit vastly different collateral circulation statuses, resulting in distinct responses to reperfusion therapy and divergent clinical outcomes; the OCSP classification fails to resolve these fine pathophysiological distinctions. Furthermore, unlike emerging biomarkers that indicate blood-brain barrier disruption or inflammatory responses (61, 62), the OCSP classification provides no insight into post-stroke microscopic pathological processes. Consequently, its predictive value for malignant cerebral edema, hemorrhagic transformation, and stroke progression is inferior to multimodal assessment models that incorporate biomarkers.

In conclusion, while the OCSP classification remains an effective clinical tool for the rapid identification of stroke types, it has distinct limitations in guiding precise reperfusion therapy and conducting refined prognostic assessments. Modern stroke management workflows should regard the OCSP classification as a preliminary anatomical framework, deeply integrating it with necessary neuroimaging technologies and biomarker detection. This approach compensates for the classification's deficiencies, ultimately enabling precise stratification and individualized treatment for stroke patients.

## 7 Conclusion and future directions

The OCSP classification provides critical clinical utility in the diagnosis, management, and prognostic assessment of complications in hyperacute cerebral infarction. By leveraging a streamlined clinical neurological evaluation, this system enables the rapid and accurate stratification of stroke subtypes. It serves not only as a guide for reperfusion therapy selection but also as a predictive tool for stroke-associated complications.

Moving forward, the synergistic integration of the OCSP classification with advanced neuroimaging and automated classification tools promises to further refine the precision of therapeutic decision-making.
